# Retrospective cohort study comparing the risk of severe hepatotoxicity in hospitalized patients treated with echinocandins for invasive candidiasis in the presence of confounding by indication

**DOI:** 10.1186/s12879-018-3333-0

**Published:** 2018-08-29

**Authors:** Francis Vekeman, Lisa Weiss, Jalal Aram, Raluca Ionescu-Ittu, Shahrzad Moosavi, Yongling Xiao, Wendy Y. Cheng, Rachel H. Bhak, Margaret Tawadrous, M. Rita Capparella, Philippe Montravers, Mei Sheng Duh

**Affiliations:** 1 Analysis Group, Inc., 111 Huntington Avenue, 14th Floor, Boston, MA 02199 USA; 2 Pfizer, Inc., New York, NY USA; 3 Pfizer PFE, Paris, France; 4 Paris Diderot Sorbonne Cite University and Bichat-Claude Bernard University Hospital, Paris, France; 5 Harvard T.H. Chan School of Public Health, Boston, MA USA

**Keywords:** Echinocandin, Anidulafungin, Caspofungin, Micafungin, Severe hepatotoxicity

## Abstract

**Background:**

To compare the risk of severe hepatotoxicity with anidulafungin versus caspofungin and micafungin in hospitalized adults.

**Methods:**

This retrospective cohort study combined data from two large US- based hospital electronic medical record databases. Severe hepatotoxicity was a Grade ≥ 3 liver function test (LFT) post-echinocandin initiation. Adjusted incidence rate ratios (IRRs) were estimated for anidulafungin versus caspofungin and micafungin, overall and in patients with normal baseline LFT (Grade 0).

**Results:**

Treatments included anidulafungin (*n* = 1700), caspofungin (*n* = 4431), or micafungin (*n* = 6547). The proportions with LFT Grade ≥ 3 pre-echinocandin initiation were: anidulafungin 40.4% versus caspofungin 25.9% (*p* <  0.001) and micafungin 25.6% (*p* <  0.001). Rates of severe underlying diseases or comorbidities were: critical care admissions: 75.3% versus 52.6 and 48.6%; and organ failures: 69.4% versus 46.7 and 51.5%. Adjusted IRRs of severe hepatotoxicity for anidulafungin versus caspofungin and micafungin were 1.43 (*p* = 0.002) and 1.19 (*p* = 0.183) overall, and 0.88 (*P* = 0.773) and 0.97 (*P* = 0.945) for normal baseline LFT, respectively.

**Conclusions:**

Accounting for confounders, severe hepatotoxicity risk was not significantly different across echinocandins in this real-world head-to-head study. Anidulafungin was used more frequently in patients with more comorbidities. Those with normal baseline LFT (least susceptible to confounding by indication), showed no elevated hepatotoxicity risk for anidulafungin.

## Background

Echinocandins are a recent class of antifungals indicated for the treatment of nosocomial infections, including candidemia and invasive candidiasis, which are associated with significant morbidity, mortality, and cost burden [1–4]. Currently marketed echinocandins in the United States (US) and Europe include anidulafungin, caspofungin, and micafungin. Compared with older generations of antifungals (i.e., amphotericin B, itraconazole, fluconazole, and voriconazole), echinocandins have been shown to have more favorable safety profiles, while having similar efficacy profiles [2–5].

According to pharmacokinetic data, anidulafungin is the only echinocandin that undergoes elimination by chemical degradation and non-specific peptidases in the plasma, instead of being metabolized by the liver [6, 7]. It is, therefore, expected that anidulafungin may lead to a lower risk of liver injuries than the other echinocandins. Nonetheless, isolated cases of severe hepatotoxicity have been noted in post-marketing spontaneous reports among patients treated with anidulafungin [8]. One possible explanation may be a result of channeling bias in clinical practice; as anidulafungin is expected to have minimal impact on the liver, physicians may preferentially use anidulafungin in more severely ill patients including those with liver impairment [9]. However, the extent of this channeling bias in the real world is unknown.

In the absence of head-to-head comparisons of anidulafungin versus other echinocandins, currently available information on the safety of anidulafungin as compared with other echinocandins is mostly derived from studies without a comparison group or from studies where anidulafungin was directly compared with antifungals other than echinocandins [10, 11]. The exception is one small study that compared outcomes between 63 critically ill patients with invasive candidiasis treated in real-world practice with anidulafungin and micafungin [12]. The study found no difference between the treatment groups in survival after adjustment for covariates, but did not investigate the risk of hepatotoxicity in multivariate analyses. To the extent liver function information is available, observational studies have the potential to rapidly collect real-world data on a large and diverse population of echinocandin users.

The present study compared the risk of severe hepatotoxicity of anidulafungin with caspofungin and micafungin among hospitalized adult patients in a real-world setting. With extensive availability of data on patients’ underlying liver function and other clinical characteristics, this study assessed the use of anidulafungin in the real world.

## Methods

### Data source

The study sample was derived from two large electronic medical record (EMR) databases, Humedica (years 2007–2013) and Cerner Health Facts (“Cerner”; years 2006–2013), the source data of which were from multiple care-delivery sites, including hospitals, large multi- specialty practices, group practices, and physician offices across all census regions of the US. The Humedica and Cerner databases were combined to obtain sufficiently large cohorts and to increase the statistical power of the study. Both databases contained information on demographic characteristics, medical history and diagnoses, detailed area of care during hospitalization (e.g., intensive care unit [ICU], critical care unit [CCU], emergency room [ER], ward, etc.), in-hospital procedures, inpatient medications including injectable and oral medications, and laboratory data (including date and time of test, and result value). To comply with Health Insurance Portability and Accountability Act guidelines, the data were de-identified by Humedica and Cerner prior to transmission to the authors for analysis. Finally, approval was obtained from the New England Institutional Review Board.

### Study design

This study employed a retrospective cohort design. The study sample included adults treated with echinocandins in hospital intensive care or inpatient settings. For patients with more than one hospitalization with echinocandin treatment, the most recent hospitalization was included in the analysis. The date of the echinocandin treatment initiation during the hospitalization was defined as the index date. The baseline period was defined as the time interval between the hospital admission date and the index date, inclusive, while the observation period was the time after the index date until the earliest event of severe hepatotoxicity (defined in Section “Liver function assessment”), hospital discharge, or death.

### Study sample inclusion and exclusion criteria

Criteria for inclusion in the study sample were: ≥18 years of age at the time of hospital admission; receipt of at least one intravenous dose of anidulafungin, caspofungin, or micafungin during hospitalization for treatment; at least one liver function test (LFT) plasma measurement (i.e., aspartate transaminase (AST), alanine aminotransferase (ALT), or total bilirubin) during the baseline period; and at least one LFT measurement during the observation period. Patients receiving more than one type of echinocandin during the hospitalization were excluded.

### Measures

#### Liver function assessment

Liver function was measured by assigning a grade of 0–4 to each type of LFT (AST, ALT, and bilirubin; Table 1) using cut-offs adapted from the Clinical Islet Transplantation study–Terminology Criteria for Adverse Events in trials of adult pancreatic islet transplantation (CIT-TCAE) Version 5.0 [13], which are modified standards of those set forth in the National Cancer Institute Common Terminology Criteria for Adverse Events (NCI-CTCAE) [14].

**Table 1:** Hepatotoxicity grade by liver function test

Liver function test	Hepatotoxicity grade^a^				
	Grade 0	Grade 1	Grade 2	Grade 3	Grade 4
Aspartate transaminase	AST ≤ ULN	AST > ULN and AST ≤ 2.5 x ULN	AST > 2.5 x ULN and AST ≤ 5 x ULN	AST > 5 x ULN and AST < 20 x ULN	AST ≥ 20 x ULN
Alanine aminotransferase	ALT ≤ ULN	ALT > ULN and ALT ≤2.5 x ULN	ALT > 2.5 x ULN and ALT ≤5 x ULN	ALT > 5 x ULN and ALT < 20 x ULN	ALT ≥20 x ULN
Total bilirubin	Bilirubin ≤ ULN	Bilirubin > ULN and bilirubin ≤1.5 x ULN	Bilirubin > 1.5 x ULN and bilirubin ≤3 x ULN	Bilirubin > 3 x ULN and bilirubin ≤10 x ULN	Bilirubin > 10 x ULN

*Abbreviations:* *ALT* alanine aminotransferase, *AST* aspartate transaminase, *ULN* upper limit of the normal

^a^Hepatotoxicity cut-offs were adapted from the Clinical Islet Transplantation study–Terminology Criteria for Adverse Events in trials of adult pancreatic islet transplantation (CIT-TCAE) Version 5.0 [13], which are modified standards of those set forth in the National Cancer Institute Common Terminology Criteria for Adverse Events (NCI-CTCAE) [14]

Overall hepatotoxicity grades were defined based on the highest grade of AST, ALT, or bilirubin tests during the baseline period to measure baseline liver function, and during the observation period to define study outcome. For the definitions of overall hepatotoxicity Grades 4 and 5, additional conditions were imposed. Specifically, overall hepatotoxicity Grade 4 was defined as having either (a) a plasma bilirubin test of Grade 4 or (b) an AST or ALT test of Grade 4 together with a diagnosis of fulminant hepatic failure (9th International Classification of Disease, Clinical Modification [ICD-9-CM] diagnosis code 572.2) and an international normalized ratio (INR) value of 2.5 or larger. Because cause of death and primary or secondary discharge diagnoses were not available in both databases, overall hepatotoxicity of Grade 5 (i.e., death due to hepatic causes) was defined in the current study as death preceded by a Grade 4 LFT.

#### Severe hepatotoxicity

The study outcome was defined as the first severe hepatotoxicity event in the observation period, regardless of whether there was any known etiology. A hepatotoxicity event was considered to be severe if it had an overall hepatotoxicity Grade of 3, 4, or 5.

#### Exposure to echinocandins

Exposure to echinocandins in a curative context was determined based on the first recorded echinocandin administration during the hospitalization, identified using the Healthcare Common Procedure Coding System (HCPCS) and National Drug Codes (NDC) as follows: anidulafungin (HCPCS code J0348 or NDC codes 00049011428, 00049011528, 00049011628, 00049101028); caspofungin (HCPCS code J0637 or NDC codes 00006382210, 00006382310); and micafungin (HCPCS code J2248 or NDC codes 00469321110, 00469325010).

#### Other covariates

Potential confounders were selected *a priori* based on published literature. Baseline liver function, defined in Section “Liver function assessment”, was expected to be the strongest confounder in the study because anidulafungin is the only echinocandin that is not metabolized by the liver and physicians may tend to prescribe it to patients with impaired liver function [7]. Additional potential confounders included patient demographics (i.e., age, gender, race), source data (i.e., Humedica, Cerner), admission through the ER, use of other non-echinocandin antifungal agents, prior use of in-hospital echinocandins, number of distinct candidiasis ICD-9-CM diagnosis codes involving different organs, Charlson comorbidity index (CCI) [15, 16], specific comorbid conditions (i.e., diabetes, endocarditis, esophageal varices, gastroesophageal reflux disease, hypertension, liver disease secondary to biliary pathologies, other liver disease, neutropenia, organ [heart or kidney] failure, and sepsis or septic shock assessed through corresponding ICD- 9-CM diagnosis codes in the administrative records of each patient; and renal dysfunction measured from estimated glomerular filtration rate laboratory test results available in the laboratory database), risk factors for fungal infections that may also be associated with higher mortality risk (i.e., admission to the ICU or CCU evaluated from place-of-service codes in the database; and use of central venous catheter [1, 7], and any type of major surgery assessed from ICD-9-CM procedure codes and/or Current Procedural Terminology codes in patients’ records), and pharmacologic treatments with known hepatotoxicity [17, 18]. In addition, given that hospital formularies may have a confounding effect through their influence on echinocandin treatment, a proxy measure of hospital formulary for echinocandins was generated based on the types of echinocandins observed in the data for a given hospital or hospital grouping in the year prior to the patient’s hospital admission.

All confounders were measured during the baseline period. However, because diagnoses made during a hospitalization could not be linked to specific dates in the Cerner data, the assessment of variables requiring diagnosis codes in Cerner data were based on diagnoses recorded at any time during the index hospitalization. For consistency, similar criteria were used for the assessment of variables requiring diagnosis codes in Humedica data.

Patients’ recovery status at the time of hospital discharge was also investigated to measure the outcomes of the hospitalizations associated with severe hepatotoxicity. Patients’ recovery status was based on reasons for patients’ discharge that were available in both Cerner and Humedica databases and included reasons such as “discharged to home”, “discharged to hospice”, or “transferred to another institution”. Two medical doctors (JA, MT) who were blinded to the treatment groups independently reviewed the list of reasons and grouped them into three categories: “recovered”, “not recovered”, or “unknown/missing”.

### Statistical analyses

To assess the association between anidulafungin and severe hepatotoxicity, separate sets of analyses were conducted for anidulafungin versus caspofungin and for anidulafungin versus micafungin. Descriptive analyses were conducted to compare the distribution of potential confounders between patients treated with anidulafungin versus those treated with micafungin or caspofungin, and to describe the demographic and clinical characteristics of patients in the three echinocandin groups at baseline. Categorical variables were compared between the echinocandin groups using chi-squared tests or Fisher exact tests (as appropriate), while continuous variables were compared between the echinocandin groups using non-parametric Wilcoxon rank-sum tests.

To account for differences in the observation period across patients, the incidence rate of severe hepatotoxicity was calculated for each echinocandin group as the number of patients with severe hepatotoxicity divided by the total person-days of observation in that group. The incidence rates were compared between anidulafungin and caspofungin or micafungin separately using univariate and multivariate negative binomial regression models. Eight covariates were forced in all models based on their clinical relevance and expected confounding effect on the choice of echinocandin treatment and/or risk of elevated LFT after the initiation of treatment.

They included baseline AST, ALT, and bilirubin grade; CCI; age; gender; dataset; and echinocandin drug formulary. Additional adjustment variables for the models were selected from the remaining covariates that were statistically associated with the choice of echinocandin treatment and also had shown statistical relevance with the outcome based on a stepwise selection methodology with significance level of 0.25 as the covariate entry cut-off and 0.10 as the covariate retaining cut-off.

### Sensitivity analyses

Three sets of sensitivity analysis were performed. The first two sets of sensitivity analysis entailed a replication of the main analyses in two subgroups: (1) the subset of patients with normal to moderately elevated LFT at baseline (Grades 0–2); and (2) the subset of patients with normal LFT at baseline (Grade 0). The third sensitivity analysis employed an alternative outcome on the full study sample, using all-cause in-hospital mortality as an outcome instead of severe hepatotoxicity. In Cerner, all-cause in-hospital death was ascertained by combining an in- hospital death indicator with information on the date of discharge. In Humedica, only an indicator for in-hospital death was available, without any information on the hospital discharge or death date for patients who died during a hospitalization. For these patients, a death date was imputed as the first day of the first gap of three or more days without any EMR data after the hospital admission.

## Results

**Fig. 1:**
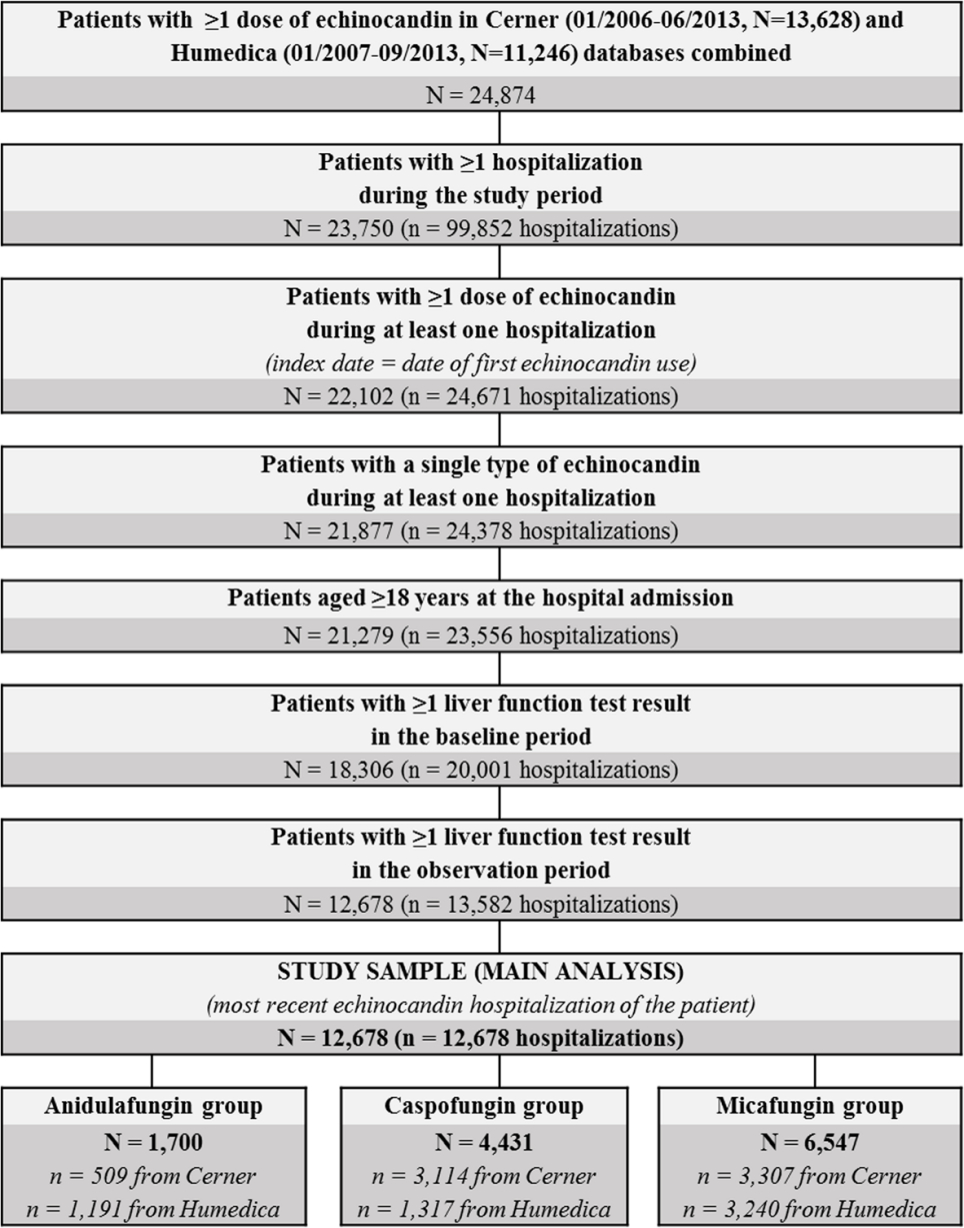
Study population selection flowchart

Of the 12,678 eligible patients in the study sample, 1,700 (13.4%) were in the anidulafungin group, 4,431 (35.0%) in the caspofungin group, and 6,547 (51.6%) in the micafungin group (Fig. 1). For the sensitivity analyses by baseline liver function subgroups, there were 9,161 (1,012 anidulafungin, 3,281 caspofungin, 4,868 micafungin) patients in the subgroup with normal to moderately elevated LFT (Grades 0–2) at baseline, of which 3,562 (320 anidulafungin, 1,207 caspofungin, 2,035 micafungin) had normal LFT at baseline (Grade 0)

### Baseline demographic and clinical characteristics

Table 2 presents patient demographic and clinical characteristics for the anidulafungin group versus the caspofungin and micafungin groups. Compared with patients in the caspofungin and micafungin groups, patients in the anidulafungin group were slightly younger (median age 60 years versus 62 and 61 years, respectively, *P* < 0.001 for both comparisons) and included more males (55.7% versus 50.1 and 51.5%, *P* < 0.001).

**Table 2:** Patient characteristics at baseline by echinocandin groups

Patient characteristics at baseline	Anidulafungin	Caspofungin	Micafungin	*P*-value	
	(*N* = 1700)	(*N* = 4431)	(*N* = 6547)	Anidulafungin versus caspofungin	Anidulafungin versus micafungin
Demographics					
Age at admission, n (%)				< 0.001*	< 0.001*
18–49 years	420 (24.7)	988 (22.3)	1528 (23.3)		
50–64 years	651 (38.3)	1463 (33.0)	2234 (34.1)		
65+ years	629 (37.0)	1980 (44.7)	2785 (42.5)		
Male, n (%)	947 (55.7)	2221 (50.1)	3371 (51.5)	< 0.001*	0.002*
Race, n (%)				< 0.001*	< 0.001*
Caucasian	988 (58.1)	3205 (72.3)	4912 (75.0)		
Black or African American	469 (27.6)	844 (19.0)	1120 (17.1)		
Asian	28 (1.6)	95 (2.1)	64 (1.0)		
Other/unknown	215 (12.6)	287 (6.5)	451 (6.9)		
Source dataset, n (%)				< 0.001*	< 0.001*
Cerner	509 (29.9)	3114 (70.3)	3307 (50.5)		
Humedica	1191 (70.1)	1317 (29.7)	3240 (49.5)		
Admission through ER, n (%)	221 (13.0)	1947 (43.9)	1359 (20.8)	< 0.001*	< 0.001*
Liver function^a^					
AST grade, n (%)				< 0.001*	< 0.001*
0	491 (28.9)	1717 (38.7)	2785 (42.5)		
1	490 (28.8)	1392 (31.4)	1884 (28.8)		
2	263 (15.5)	569 (12.8)	772 (11.8)		
3	248 (14.6)	468 (10.6)	673 (10.3)		
4	204 (12.0)	273 (6.2)	430 (6.6)		
Unknown	4 (0.2)	12 (0.3)	3 (0.0)		
ALT grade, n (%)				< 0.001*	< 0.001*
0	773 (45.5)	2582 (58.3)	3725 (56.9)		
1	395 (23.2)	969 (21.9)	1424 (21.8)		
2	182 (10.7)	401 (9.0)	534 (8.2)		
3	177 (10.4)	294 (6.6)	442 (6.8)		
4	127 (7.5)	144 (3.2)	269 (4.1)		
Unknown	46 (2.7)	41 (0.9)	153 (2.3)		
Total bilirubin grade, n (%)				< 0.001*	< 0.001*
0	663 (39.0)	2056 (46.4)	3653 (55.8)		
1	272 (16.0)	664 (15.0)	853 (13.0)		
2	265 (15.6)	708 (16.0)	909 (13.9)		
3	314 (18.5)	516 (11.6)	692 (10.6)		
4	156 (9.2)	174 (3.9)	279 (4.3)		
Unknown	30 (1.8)	313 (7.1)	161 (2.5)		
Overall grade of hepatotoxicity, n (%)				< 0.001*	< 0.001*
Grade 0	320 (18.8)	1207 (27.2)	2035 (31.1)		
Grade 1	390 (22.9)	1182 (26.7)	1693 (25.9)		
Grade 2	302 (17.8)	892 (20.1)	1140 (17.4)		
Grade 3	524 (30.8)	971 (21.9)	1395 (21.3)		
Grade 4	164 (9.6)	179 (4.0)	284 (4.3)		
Fungal infection					
Prior use of in-hospital echinocandin, n (%)	106 (6.2)	232 (5.2)	390 (6.0)	0.125	0.667
Candidiasis diagnosis^b,^^c^, n (%)	574 (33.8)	877 (19.8)	1307 (20.0)	< 0.001*	< 0.001*
Number of distinct candidiasis ICD-9-CM diagnosis codes, n (% patients with a candidiasis diagnosis)				< 0.001*	< 0.001*
1	382 (66.6)	760 (86.7)	1130 (86.5)		
2	158 (27.5)	107 (12.2)	159 (12.2)		
≥ 3	34 (5.9)	10 (1.1)	18 (1.4)		
Comorbidities^c^					
CCI^d^, n (%)				< 0.001*	< 0.001*
0	268 (15.8)	1473 (33.2)	1759 (26.9)		
1	182 (10.7)	551 (12.4)	832 (12.7)		
2	223 (13.1)	798 (18.0)	1221 (18.6)		
3	219 (12.9)	530 (12.0)	875 (13.4)		
≥ 4	808 (47.5)	1079 (24.4)	1860 (28.4)		
Specific comorbidities, n (%)					
Liver diseases					
Esophageal varices	41 (2.4)	42 (0.9)	83 (1.3)	< 0.001*	< 0.001*
Liver disease secondary to biliary pathologies^e^	275 (16.2)	330 (7.4)	648 (9.9)	< 0.001*	< 0.001*
Other liver disease^f^	217 (12.8)	322 (7.3)	629 (9.6)	< 0.001*	< 0.001*
Other comorbidities					
Diabetes	477 (28.1)	848 (19.1)	1489 (22.7)	< 0.001*	< 0.001*
Endocarditis	168 (9.9)	89 (2.0)	185 (2.8)	< 0.001*	< 0.001*
Gastroesophageal reflux disease	147 (8.6)	386 (8.7)	704 (10.8)	0.936	0.011*
Hypertension	913 (53.7)	1292 (29.2)	2325 (35.5)	< 0.001*	< 0.001*
Neutropenia	145 (8.5)	354 (8.0)	542 (8.3)	0.489	0.739
Organ failures	1179 (69.4)	2071 (46.7)	3372 (51.5)	< 0.001*	< 0.001*
Sepsis or septic shock	1165 (68.5)	2080 (46.9)	3137 (47.9)	< 0.001*	< 0.001*
Renal dysfunction (CKD stage)^g^				< 0.001*	< 0.001*
Stage 1 (GFR ≥ 90 mL/min/1.73 m)	154 (9.1)	411 (9.3)	729 (11.1)		
Stage 2 (GFR 60–89)	240 (14.1)	898 (20.3)	1363 (20.8)		
Stage 3 (GFR 30–59)	482 (28.4)	1282 (29.0)	1786 (27.3)		
Stage 4 (GFR 15–29)	401 (23.6)	953 (21.5)	1395 (21.3)		
Stage 5 (GFR < 15)	423 (24.9)	883 (19.9)	1271 (19.4)		
Risk factors for fungal infection, n (%)					
Admission to ICU or CCU	1280 (75.3)	2330 (52.6)	3184 (48.6)	< 0.001*	< 0.001*
Use of central venous catheter	744 (43.8)	590 (13.3)	1262 (19.3)	< 0.001*	< 0.001*
Surgery	698 (41.1)	1492 (33.7)	1773 (27.1)	< 0.001*	< 0.001*
Hospital formulary proxy, n (%)				< 0.001*	< 0.001*
All three echinocandins covered	358 (21.1)	1243 (28.1)	1797 (27.4)		
Anidulafungin and caspofungin covered	527 (31.0)	1322 (29.8)	0 (0.0)		
Anidulafungin and micafungin covered	216 (12.7)	0 (0.0)	350 (5.3)		
Caspofungin and micafungin covered	0 (0.0)	330 (7.4)	2742 (41.9)		
Single agent covered	599 (35.2)	1536 (34.7)	1658 (25.3)		
Hepatotoxic drugs initiated in the baseline period^h^					
Number of distinct hepatotoxic drugs, median [IQR]	12.0 (8.0, 16.0)	12.0 (8.0, 15.0)	12.0 (9.0, 17.0)	< 0.001*	0.123
Acetaminophen, n (%)	1255 (73.8)	3629 (81.9)	5373 (82.1)	< 0.001*	< 0.001*
Antibiotics, n (%)	785 (46.2)	2384 (53.8)	3357 (51.3)	< 0.001*	< 0.001*
Antidiabetics, n (%)	22 (1.3)	96 (2.2)	98 (1.5)	0.026*	0.534
Antimycobacterials, n (%)	58 (3.4)	116 (2.6)	125 (1.9)	0.094	< 0.001*
Antiretrovirals, n (%)	40 (2.4)	38 (0.9)	56 (0.9)	< 0.001*	< 0.001*
Chemotherapies, n (%)	87 (5.1)	271 (6.1)	401 (6.1)	0.136	0.117
Non-steroidal anti-inflammatory drug, n (%)	622 (36.6)	1567 (35.4)	2456 (37.5)	0.371	0.482
Psychotropics, n (%)	484 (28.5)	1379 (31.1)	1917 (29.3)	0.043*	0.512

*P-value < 0.05

*Abbreviations: ALT* alanine aminotransferase, *AST* aspartate transaminase, *CCI* Charlson comorbidity index, *CCU* critical care unit, *CKD* chronic kidney disease, *ER* emergency room, *GFR* glomerular filtration rate, *ICD-9-CM* 9th International Classification of Disease, Clinical Modification, *ICU* intensive care unit, *IQR* inter-quartile range

^a^Patients did not necessarily have all the three liver function tests (AST, ALT, total bilirubin), but they had at least one

^b^We expect candidiasis was the indication for echinocandin treatment for most patients; possibly undercoded in the hospitalization records

^c^Defined based on ICD-9-CM diagnosis codes. Since diagnosis dates werenot available in the Cerner database, diagnoses were measured over the entire duration of the hospitalization

^d^CCI, an index that was developed to predict one-year mortality in hospitalized patients; CCI is calculated based on the presence of ICD-9-CM diagnosis codes for 17 comorbidities associated with high risk of death, such as cancer, myocardial infarction, congestive heart failure, diabetes, and others; values range from 0 to 33, with higher values indicating higher risk of death

^e^Includes cholelithiasis and other disorders of gallbladder (e.g., acute cholecystitis, obstruction of gallbladder, fistula of gallbladder) or biliary tract (e.g., postcholecystectomy syndrome cholangitis, obstruction of bile duct)

^f^Includes severe forms of viral hepatitis, acute and subacute necrosis of liver, chronic liver disease, cirrhosis, and liver transplant

^g^ Levey AS, Coresh J, Balk E, Kausz AT, Levin A, Steffes MW, Hogg RJ, Perrone RD, Lau J, Eknoyan G et al. National Kidney Foundation practice guidelines for chronic kidney disease: evaluation, classification, and stratification. Ann Intern Med 2003;139(2):137–147

^h^Hepatotoxic medications, based on the literature, were identified using National Drug Codes (NDC) and classified using Generic Product Identifier (GPI) and American Hospital Formulary Service (AHFS) categories; these medications include acetaminophen and selected drugs in the following classes: antibiotics, antidiabetics, antimycobacterials, antiretrovirals, chemotherapies, non-steroidal anti-inflammatory drugs, and psychotropics

All measurements of LFT parameters pointed to a higher degree of abnormal liver function at baseline for the anidulafungin group relative to the caspofungin and micafungin groups (with all *p*-values < 0.001). There were statistically significantly more patients with AST, ALT, and total bilirubin tests of Grades 3–4 in the baseline period in the anidulafungin group than in the caspofungin and micafungin groups (AST: 26.6% versus 16.8% and 16.9%; ALT: 17.9% versus 9.8 and 10.9%; bilirubin: 27.7% versus 15.5 and 14.9%; all *P*-values < 0.001), which translated into more patients with an overall Grade 3 and 4 for the baseline LFTs in the anidulafungin group than in the caspofungin and micafungin groups (40.4% versus 25.9 and 25.6%, *P* < 0.001 for both comparisons).

Candidiasis data based on diagnosis codes (ICD-9-CM code 112.x, which includes site- specific codes such as candidiasis of the mouth, candidiasis of skin and nails, or disseminated candidiasis, and non-specific candidiasis codes such as candidiasis or other candidiasis) were largely missing in the dataset.

Patients in the anidulafungin group had higher overall comorbidities (CCI score ≥4: 47.5% versus 24.4 and 28.4%); greater disease severity (represented by organ failures: 69.4% versus 46.7 and 51.5%; and sepsis or septic shock: 68.5% versus 46.9 and 47.9%), tended to have more risk factors for fungal infection than patients in the other echinocandin groups (critical care admissions: 75.3% versus 52.6 and 48.6%; surgeries: 41.1% versus 33.7 and 27.1%: use of central venous catheters: 43.8% versus 13.3 and 19.3%), and had more underlying liver diseases (liver diseases secondary to biliary pathology: 16.2% versus 7.4 and 9.9%; other liver disease [defined in Table 2 footnote]: 12.8% versus 7.3 and 9.6%; *P* < 0.001 for all). The median number of distinct hepatotoxic drugs (as defined in Table 2) used during the baseline period was similar for all three echinocandin groups.

### Severe hepatotoxicity outcome

In the anidulafungin, caspofungin, and micafungin groups, 37.2, 22.4, and 23.3% of patients experienced a severe hepatotoxicity event over a median observation period of 9, 12, and 10 days, respectively (Table 3). Among patients receiving anidulafungin, caspofungin, and micafungin, Grade 3 events were the first severe hepatotoxicity events for 29.6, 19.6, and 19.7%; and Grade 4 events were the first severe hepatotoxicity events for 7.6, 2.8, and 3.6%, respectively. Among patients who experienced a severe hepatotoxicity event, there were no differences between anidulafungin, caspofungin, and micafungin in the overall proportion of patients with a discharge status of “not recovered” (anidulafungin 66.8% versus caspofungin 64.8% versus micafungin 65.0%) or with a discharge status of “recovered” (anidulafungin 31.3% versus caspofungin 31.8% [ *p* = 0.175] versus micafungin 32.3% [*P* = 0.470] ) (Table 3).

**Table 3:** Outcome distribution among the three echinocandin groups

	Anidulafungin	Caspofungin	Micafungin
Number of patients, n	1700	4431	6547
Patient-days in observation period			
Total person-days	26,449	85,020	102,267
Distribution person-days across patients (all patients)			
Median [IQR]	9.0 (2.0, 20.0)	12.0 (5.0, 23.0)	10.0 (4.0, 20.0)
Mean ± SD [Range]	15.6 ± 23.3 (1–309)	19.2 ± 31.7 (1–938)	15.6 ± 20.0 (1–350)
Severe hepatotoxicity events^a^			
Patients with severe hepatotoxicity events, n (% of all patients)	633 (37.2)	993 (22.4)	1522 (23.2)
Grade of first severe hepatotoxicity event in the observation period, n (% of all patients)			
Grade 3	504 (29.6)	870 (19.6)	1289 (19.7)
Grade 4	129 (7.6)	123 (2.8)	233 (3.6)
Grade 5^b^	–	–	–
Status at hospital discharge, n (% of patients with events)			
Recovered	198 (31.3)	316 (31.8)	491 (32.3)
Not recovered	423 (66.8)	643 (64.8)	990 (65.0)
Unknown	12 (1.9)	34 (3.4)	41 (2.7)

*Abbreviations: IQR* inter-quartile range, *SD* standard deviation

^a^A severe hepatotoxic event was defined as the first hepatotoxicity of Grade ≥ 3 in the observation period. Hepatotoxicity grade was classified according to the modified Clinical Islet Transplantation study–Terminology Criteria for Adverse Events in trials of adult pancreatic islet transplantation (CIT-TCAE) Version 5.0 [13]

^b^Grade 5 events cannot be the first event experienced by the patient because Grade 5 events (deaths) must be preceded, by definition, by a liver function test of Grade 4

The crude incidence rates of severe hepatotoxicity per month in the anidulafungin, caspofungin, and micafungin groups were 0.72, 0.35, and 0.45, respectively, corresponding to the unadjusted incidence rate ratios (IRRs) of 2.05 (95% confidence interval [CI] 1.85, 2.26) for the comparisons of anidulafungin versus caspofungin and 1.61 (95% CI 1.47, 1.76), for the comparisons of anidulafungin versus micafungin. After adjusting for the baseline LFT and other measured confounders, the IRR for anidulafungin versus caspofungin decreased to 1.43 (95% CI 1.14, 1.79; *P* = 0.002) and remained statistically significant, while the IRR for anidulafungin versus micafungin decreased to 1.19 (95% CI 0.92, 1.54; *P* = 0.183) and was not statistically significant (Table 4).

**Table 4:** Incidence rates and adjusted incidence rate ratios of severe hepatotoxicity: main analysis and sensitivity analysis

	Total person-days			Number of outcome events (incidence rate per 30 person-days)			Adjusted IRR (95% CI)*P*-values	
	Anidulafungin	Caspofungin	Micafungin	Anidulafungin	Caspofungin	Micafungin	Anidulafungin versus caspofungin	Anidulafungin versus micafungin
Main analysis								
Sample: all patients Outcome: severe hepatotoxicity	26449	85020	102267	633 (0.72)	993 (0.35)	1522 (0.45)	1.43 (1.14, 1.79)* 0.002*	1.19 (0.92, 1.54) 0.183
Sensitivity analyses								
Subgroup: patients with normal LFT at baseline (Grade 0) Outcome: severe hepatotoxicity	6538	23703	33926	26 (0.12)	67 (0.08)	131 (0.12)	0.88 (0.36, 2.14) 0.773	0.97 (0.46, 2.08) 0.945
Subgroup: patients with normal to moderately elevated LFT at baseline (Grades 0–2) Outcome: severe hepatotoxicity	20102	69188	84415	154 (0.23)	343 (0.15)	513 (0.18)	1.46 (0.91, 2.37) 0.119	1.62 (0.95, 2.77) 0.078
Sample: all patients Outcome: all-cause in-hospital death	38246	104801	126348	581 (0.46)	1188 (0.34)	1544 (0.37)	1.26 (1.07, 1.48)* 0.007*	0.93 (0.77, 1.12) 0.444

*P-value < 0.05

*Abbreviations: CI* confidence interval, *IRR* incidence rate ratio, *LFT* liver function test

The multivariate regression models from which the adjusted IRRs for echinocandin treatments were obtained are presented in Table 5 and show that, for both models, the strongest predictor of severe hepatotoxicity was baseline abnormal LFT (bilirubin of Grades > 0, baseline AST of Grades > 0, baseline ALT of Grades > 2). The other strong predictors of severe hepatotoxicity were: presence of other liver disease (other than liver diseases secondary to biliary pathology), esophageal varices, and sepsis for the anidulafungin versus caspofungin model; and esophageal varices, presence of other liver disease, chronic kidney disease stage 4, CCI ≥ 3, and sepsis for the anidulafungin versus micafungin model.

**Table 5:** Adjusted incidence rate ratios of severe hepatotoxicity – final model for main analysis

	Patients treated with anidulafungin versus caspofungin (*N* = 6131)		Patients treated with anidulafungin versus micafungin (*N* = 8247)	
	IRR (95% CI)	*P*-value	IRR (95% CI)	*P*-value
Echinocandin exposure				
Anidulafungin versus caspofungin	1.43 (1.14, 1.79)*	0.0022*	–	–
Anidulafungin versus micafungin	–	–	1.19 (0.92, 1.54)	0.1825
Demographics				
Age categories (ref: 18–49 years)				
50–64 years	1.06 (0.87, 1.30)	0.5517	1.06 (0.89, 1.25)	0.5101
65+ years	1.10 (0.90, 1.35)	0.3317	1.14 (0.96, 1.36)	0.1245
Female (ref: male)	0.91 (0.79, 1.07)	0.2511	0.97 (0.86, 1.11)	0.6880
Cerner dataset (ref: Humedica)	0.76 (0.58, 1.00)*	0.0466*	0.82 (0.69, 0.99)*	0.0379*
Admission through ER	0.80 (0.67, 0.96)*	0.0151*	0.77 (0.64, 0.92)*	0.0053*
Baseline liver function				
ALT grade (ref: 0)^a^				
1	1.13 (0.91, 1.41)	0.2746	1.13 (0.93, 1.37)	0.2236
2	1.17 (0.85, 1.62)	0.3291	1.31 (1.00, 1.72)*	0.0484*
3	1.91 (1.29, 2.82)*	0.0013*	1.71 (1.24, 2.37)*	0.0012*
4	2.38 (1.40, 4.05)*	0.0014*	2.34 (1.53, 3.58)*	< 0.0001*
AST grade (ref: 0)^a^				
1	1.75 (1.40, 2.20)*	< 0.0001*	1.96 (1.61, 2.39)*	< 0.0001*
2	2.24 (1.66, 3.02)*	< 0.0001*	2.63 (2.02, 3.41)*	< 0.0001*
3	3.90 (2.73, 5.57)*	< 0.0001*	4.67 (3.45, 6.33)*	< 0.0001*
4	4.62 (2.79, 7.63)*	< 0.0001*	6.89 (4.56, 10.4)*	< 0.0001*
Bilirubin grade (ref: 0)^a^				
1	1.71 (1.33, 2.19)*	< 0.0001*	1.63 (1.32, 2.01)*	< 0.0001*
2	2.84 (2.24, 3.60)*	< 0.0001*	2.77 (2.28, 3.36)*	< 0.0001*
3	13.8 (10.9, 17.3)*	< 0.0001*	12.1 (9.96, 14.7)*	< 0.0001*
4	25.9 (19.3, 34.7)*	< 0.0001*	17.2 (13.3, 22.1)*	< 0.0001*
Fungal infection				
Prior use of in-hospital echinocandin	–	–	1.31 (0.99, 1.74)	0.0567
Number of fungal infection sites (ref: 1)				
≥ 2	0.81 (0.54, 1.20)	0.2935	0.85 (0.61, 1.19)	0.3479
No candidiasis ICD-9-CM codes	1.59 (1.29, 1.95)*	< 0.0001*	1.36 (1.14, 1.61)*	0.0005*
Comorbidities				
Charlson Comorbidity Index (ref: 0)				
1	0.79 (0.59, 1.06)	0.1111	0.77 (0.60, 1.00)	0.0545
2	0.80 (0.61, 1.04)	0.0990	0.99 (0.78, 1.25)	0.9042
3	0.75 (0.56, 1.01)	0.0599	1.28 (1.00, 1.65)*	0.0496*
≥ 4	1.17 (0.90, 1.52)	0.2497	1.41 (1.12, 1.78)*	0.0036*
Liver diseases				
Esophageal varices	1.83 (1.10, 3.05)*	0.0194*	1.65 (1.11, 2.46)*	0.0130*
Liver disease secondary to biliary pathologies	1.15 (0.91, 1.45)	0.2427	1.18 (0.98, 1.42)	0.0768
Other liver disease	1.96 (1.53, 2.51)*	< 0.0001*	1.52 (1.26, 1.83)*	< 0.0001*
Other comorbidities				
Diabetes	0.67 (0.55, 0.83)*	0.0002*	0.90 (0.76, 1.06)	0.2073
Endocarditis	0.59 (0.40, 0.87)*	0.0073*	–	–
Gastroesophageal reflux disease	0.83 (0.62, 1.11)	0.2089	0.90 (0.72, 1.13)	0.3778
Hypertension	0.72 (0.59, 0.89)*	0.0022*	0.78 (0.67, 0.92)*	0.0022*
Neutropenia	–	–	1.26 (1.00, 1.59)*	0.0470*
Organ failures	1.21 (0.99, 1.47)	0.0602	1.18 (0.98, 1.42)	0.0756
Sepsis	1.46 (1.22, 1.75)*	< 0.0001*	1.34 (1.15, 1.56)*	0.0001*
Renal dysfunction (CKD stage) (ref: Stage 1 [GFR ≥ 90 mL/min/1.73 m])				
Stage 2 (GFR 60–89)	–	–	1.21 (0.91, 1.63)	0.1915
Stage 3 (GFR 30–59)	–	–	1.24 (0.93, 1.64)	0.1378
Stage 4 (GFR 15–29)	–	–	1.53 (1.14, 2.06)*	0.0047*
Stage 5 (GFR < 15)	–	–	1.07 (0.79, 1.45)	0.6534
Risk factors for fungal infection				
Admission to ICU or CCU	–	–	1.32 (1.15, 1.53)*	0.0001*
Use of central venous catheter	–	–	0.73 (0.62, 0.87)*	0.0003*
Surgery	0.63 (0.53, 0.74)*	< 0.0001*	0.66 (0.57, 0.76)*	< 0.0001*
Hospital formulary proxy (ref: anidulafungin only covered)				
All three echinocandins covered	0.97 (0.71, 1.33)	0.8532	1.17 (0.85, 1.60)	0.3382
Anidulafungin and caspofungin covered	0.95 (0.69, 1.29)	0.7249	0.93 (0.68, 1.27)	0.6426
Anidulafungin and micafungin covered	1.22 (0.79, 1.87)	0.3701	1.12 (0.80, 1.58)	0.5090
Caspofungin and micafungin covered	0.95 (0.59, 1.52)	0.8169	1.01 (0.71, 1.44)	0.9584
Caspofungin only covered	1.11 (0.76, 1.62)	0.5756	–	–
Micafungin only covered	–	–	1.00 (0.69, 1.44)	0.9910
Hepatotoxic drugs initiated in the baseline period				
Number of distinct hepatotoxic drugs (ref: 0–5)				
6–10	–	–	0.73 (0.57, 0.93)*	0.0095*
11–25	–	–	0.58 (0.46, 0.73)*	< 0.0001*
26–48	–	–	0.42 (0.25, 0.69)*	0.0006*
Acetaminophen	–	–	0.82 (0.70, 0.96)*	0.0135*
Antibiotics	–	–	0.94 (0.83, 1.07)	0.3741

*P-value < 0.05

*Abbreviations: ALT* alanine aminotransferase, *AST* aspartate transaminase, *CI* confidence interval, *CCU* critical care unit, *CKD* chronic kidney disease, *ER* emergency room, *GFR* glomerular filtration rate, *ICD-9-CM* 9th International Classification of Disease, Clinical Modification, *ICU* intensive care unit, *IRR* incidence rate ratio

^a^Unknown categories not shown

### Sensitivity analyses

In the subgroup of patients with normal LFT at baseline, 12% of those treated with anidulafungin, 8% of those treated with caspofungin, and 12% of those treated with micafungin had experienced a hepatotoxicity event (Table 4). The results of the sensitivity analyses conducted in this subgroup showed that anidulafungin had a lower but non-statistically significantly different risk from caspofungin and micafungin (IRRs: 0.88 [95% CI 0.36, 2.14], *P* = 0.773, and 0.97 [95% CI 0.46, 2.08], *P* = 0.945 for anidulafungin versus caspofungin and anidulafungin versus micafungin, respectively) (Table 4).

In the subgroup of patients with Grades 0–2 liver toxicity (normal, mildly elevated, or moderately elevated LFT) at baseline, 23% of those treated with anidulafungin, 15% of those treated with caspofungin, and 18% of those treated with micafungin had experienced a hepatotoxicity event (Table 4). The results of the sensitivity analyses conducted in this subgroup showed that, after adjustment for baseline LFT and other potential confounders, the IRR estimates for the risk of severe hepatotoxicity were not statistically significant in either comparison (IRRs: 1.46 [95% CI 0.91, 2.37], *P* = 0.119, and 1.62 [95% CI 0.95, 2.77], *P* = 0.078 for anidulafungin versus caspofungin and anidulafungin versus micafungin, respectively) (Table 4).

The sensitivity analysis conducted in the full study sample using all-cause in-hospital death as the outcome showed similar results with those from the main analyses, but with weaker effects (adjusted IRR: 1.26 [95% CI 1.07, 1.48], *P* = 0.007, and 0.93 [95% CI 0.77, 1.12], *P* = 0.444 for anidulafungin versus caspofungin and anidulafungin versus micafungin, respectively).

## Discussion

To investigate whether anidulafungin is associated with an elevated risk of severe hepatotoxicity compared with other echinocandins in a post-marketing setting, there is a need for head-to-head studies that analyze data derived from the same source population. Furthermore, because of the metabolism pathways of anidulafungin [7], the confounding by indication phenomenon where anidulafungin is channeled towards patients at a high risk for liver injuries [9] is likely to exist in non-randomized observational clinical practice settings.

To our knowledge, this is the first large head-to-head study to compare the risk of severe hepatotoxicity of echinocandins among hospitalized adult patients. Without any statistical adjustments, the risk of severe hepatotoxicity appeared to be significantly higher in hospitalized patients treated with anidulafungin than in those treated with caspofungin or micafungin. However, this may be due to channeling of anidulafungin towards patients with impaired liver function and more severe comorbidity profiles. After adjustment for measurable confounders available in the data, the risk of severe hepatotoxicity for anidulafungin was not significantly elevated compared with caspofungin and micafungin in most analyses. Importantly, in the subgroup of patients with normal LFT at baseline, there was no evidence of elevation of severe hepatotoxicity risk for anidulafungin. While the sample size in this subgroup was relatively small and did not permit robust interpretation (hence, it was considered as a sensitivity analysis), this was the most ideal sub-population for assessing causal association between echinocandin treatments and treatment- emergent hepatotoxicity events, because it was the least susceptible for confounding by indication bias. There was no difference in the incidence rate of all-cause in-hospital death between the anidulafungin and micafungin groups. Although patients in the anidulafungin group were found to have a statistically significantly higher incidence rate of all-cause in-hospital death than patients in the caspofungin group, this significant difference was likely to be the result of confounding by indication.

Our findings are consistent with the results from previous studies. A 2016 single-center study of 63 critically ill patients treated in real-world practice with anidulafungin and micafungin found higher rates of pre-treatment liver failure for anidulafungin-treated patients as compared to micafungin-treated patients (13% vs. 0%) [12], suggesting patients with liver impairment are channeled towards anidulafungin. In the same study, patients treated with anidulafungin had higher LFT values during treatment than those treated with micafungin, but these differences disappeared when the sample was restricted to patients without liver failure, suggesting pre-treatment liver failure explained most differences in LFT [12]. Another study from 2016 found similar rates of hepatic injury in 2,970 micafungin recipients and 6,726 patients treated with other parenteral antifungal agents, including anidulafungin (13 vs. 12 events per 100 patients), after adjustment for confounding via propensity score matching [19]. Similarly, a 2010 meta-analysis of RCT and non-randomized study data found a similar or lower proportion of patients who experienced abnormal LFTs among anidulafungin users versus caspofungin and micafungin users, even though the treatment groups were not extracted from the same source population [11]. Finally, in a safety study of anidulafungin among 86 adult solid organ transplantation (SOT) recipients, only one patient developed mild liver toxicity, suggesting anidulafungin is well-tolerated drug even in this high-risk population [20]. Nevertheless, the proportion of echinocandin-treated patients who experienced severe hepatotoxicity event in the current study (22.4–37.2%) was higher than that reported in previous studies. For example, the 2010 meta-analysis of RCT and non- randomized study reported that 1.0% of the patients experienced abnormal LFTs requiring cessation of treatment and 3.8% experienced abnormal LFTs not requiring cessation of treatment [11]. Furthermore, a 2017 single-center study by Shibata et al. reported severe hepatotoxicity event rates of 6.1 - 7.4% for 201 hospitalized patients treated with caspofungin or micafungin [21]. Several factors could explain the high proportion of patients in the current study who experienced hepatotoxicity events. First, a large proportion of the patients in the current sample had liver impairment at baseline. Given that elevated LFT in the baseline period was found in the current study to be a strong predictor of severe hepatotoxicity post-treatment, higher baseline LFT is likely to translate into a higher proportion of patients with severe hepatotoxicity outcomes. Indeed, the proportion of patients with caspofungin and micafungin-treated patients with pre-treatment overall hepatotoxicity of grade 2 in the current study was about twice as large as that reported in the single-center study by Shibata et al. (43 - 46% vs. 21 - 29% [21]). Furthermore, real-world patients are usually more severely ill than those enrolled in RCTs due to the restrictive eligibility criteria for enrolment in most trials [11, 22], which could explain the differences in hepatotoxicity event rates between the current study and the 2010 meta-analysis. Second, the current study excluded patients who did not have an LFT measurement in both the baseline (14%) and observation periods (40%) (Fig. 1). If physicians are more likely to order LFTs for patients who are at risk of elevated liver enzymes, not having an LFT in the baseline period may be an indicator of a lower risk of developing hepatotoxicity.

Compared to other echinocandins, the current study showed that patients receiving anidulafungin treatment were significantly more likely to have baseline impaired liver function and more severe comorbidity profiles, as shown in CCI and rates of critical care admissions, organ failures, and sepsis and septic shock. This confounding by indication bias is well-known in epidemiology literature and adjustment is methodologically challenging [23–25]. In our case, anidulafungin is the only echinocandin, among the three studied, that is not metabolized by the liver and does not require dose adjustment in patients with severe hepatic impairment [6, 7]. Because of that, physicians in clinical practice are likely to be more inclined to use anidulafungin for patients who have a history of hepatotoxicity. A recent small retrospective study confirmed the real-world channeling bias in practice where anidulafungin was used in critically ill patients with invasive candidiasis [12]. This creates a paradoxical artifact that anidulafungin use appears to be associated with a higher risk of hepatotoxicity in the absence of any adjustment. To control for confounding by indication, we identified potential confounders at baseline and used them as adjustment variables in the analyses. The hospital EMR data source used in this study provided ample laboratory data to ascertain patients’ liver function prior to the initiation of echinocandin treatments. However, despite our best efforts to identify and control for potential confounders, residual confounding due to unobservable factors in the database may have remained.

In an effort to control for confounding by indication with respect to LFT, subgroup analyses were conducted on both patients with Grade 0 and Grades 0–2 LFT at baseline. Notably, in the subgroup of patients with normal baseline LFT, the subgroup most likely to be free of confounding by indication bias, no evidence was found for any elevated risk of severe hepatotoxicity associated with anidulafungin relative to the other echinocandins. In this subgroup analysis, residual confounding by indication due to higher comorbidity burden still exists; therefore, to the extent that comorbidities may contribute to higher risk of hepatic injuries, the true causation of anidulafungin on liver injury risk may be even lower.

Among patients who experienced a severe hepatotoxicity event in the current study, there were no differences between anidulafungin and the other echinocandins in hospital discharge status. The overall proportions of patients who “recovered” before discharge were similar among the three echinocandin groups, suggesting no significant variation in the potential recovery and sequelae of severe hepatotoxicity events across the treatment groups.

The study had several limitations. First, in the analysis on the overall sample, severe hepatotoxicity was defined as having LFT results greater than Grade 3 in the observation period regardless of patient baseline LFT results. Therefore, the outcome included not only incident cases of severe hepatotoxicity (i.e., baseline LFT was normal), but also prevalent cases. As discussed previously, given that a greater proportion of patients on anidulafungin had Grade 3 or higher LFT at baseline, the current definition would have identified more prevalent cases in the anidulafungin patients, thereby biasing the effect estimates against anidulafungin. This limitation was addressed by conducting sensitivity analyses among the subgroup of patients with normal, mildly elevated, or moderately elevated LFT results during the baseline period, but these subgroups had smaller sample sizes and reduced power. Furthermore, the study only collected data during the hospitalization period. As such, patients’ full medical history prior to the hospitalization and long-term effects of echinocandins (post-hospital discharge) were not captured. However, the risk of hepatotoxicity was likely driven more by the patients’ acute health status during the hospitalization (rather than chronic medical history) and, because intravenous echinocandins are usually used over short periods of time during hospitalization, the effect of echinocandin is more likely to be acute than chronic. Thus, we do not expect the results of the study to be impacted much by this limitation.

Due to data limitations, confounders assessed based on ICD-9-CM diagnosis codes were measured over the full hospitalization period, making it difficult to assess the temporality between these diagnoses, exposure to echinocandin, and severe hepatotoxicity. However, for chronic conditions, which comprise the majority of the diagnoses considered in this study, one can assume that they were likely pre-existing prior to echinocandin initiation rather than newly developed during the short observation period. In addition, the study population may have excluded some patients with good prognosis of severe hepatotoxicity by having restricted the analysis to patients who had at least one LFT result during the baseline and observation periods. As such, the risks observed across the echinocandins in the study may have been overestimated in all treatment groups, and the generalizability of the study population needs to be considered. However, in a sensitivity analysis that included patients without baseline LFT, the results were consistent with the main analysis (adjusted IRRs: 1.37 [95% CI 1.09, 1.71] and 1.19 [95% CI 0.92, 1.54] for anidulafungin versus caspofungin and micafungin, respectively; data not shown), suggesting that the selection of patients with baseline LFT for the main analyses had minimal impact on the generalizability of the results. Moreover, dosages and duration of echinocandins use were not available in the data and could not be assessed in this study.

Furthermore, we noted that patients receiving anidulafungin were mostly contributed by the Humedica database, while patients receiving caspofungin mostly came from the Cerner database, which may have been due to differences in treatment practices and drug formularies between the respective centers. Finally, prior to 2012 anidulafungin formulation included ethanol, which may have had an effect on hepatotoxicity among patients treated with anidulafungin . The study was also subject to general limitations intrinsic to EMR data, such as possible inaccuracies in coding diagnoses, procedures, or pharmacy orders.

## Conclusions

Based on real-world hospital practice data, most analyses from this study indicated that the risk of severe hepatotoxicity was not statistically significantly different between patients treated with anidulafungin and those treated with caspofungin or micafungin. There is a clear evidence of favoring the use of anidulafungin treatment in sicker patients with worse prognosis and comorbidities. The confounding by indication bias may not have been fully adjusted for in the analysis. In the subgroup of patients with normal baseline LFT, who were least susceptible to confounding by indication bias, anidulafungin was not associated with any elevation of risk of severe hepatotoxicity compared with caspofungin and micafungin.

## Abbreviations

ALT: Alanine aminotransferase
AST: Aspartate transaminase
CCI: Charlson comorbidity index
CCU: Critical care unit
CI: Confidence interval
CIT-TCAE: Clinical Islet Transplantation study–Terminology Criteria for Adverse Events
CKD: Chronic kidney disease
EMR: Electronic medical record
ER: Emergency room
GFR: Glomerular filtration rate
HCPCS: Healthcare Common Procedure Coding System
ICD-9-CM: 9th International Classification of Disease, Clinical Modification
ICU: Intensive care unit
INR: International normalized ratio
IQR: Inter-quartile range
IRR: Incidence rate ratios
LFT: Liver function test
NCI-CTCAE: National Cancer Institute Common Terminology Criteria for Adverse Events
NDC: National Drug Codes
SD: Standard deviation
US: United States
